# Predictors of the perceived risk of COVID-19 and adherence to confinement guidelines in the context of the COVID-19 pandemic

**DOI:** 10.1192/j.eurpsy.2021.1795

**Published:** 2021-08-13

**Authors:** M. Jarego, E. Rodríguez, A. Ciaramella, J. Miró, J. Pais-Ribeiro

**Affiliations:** 1 Health Psychology, ISPA, Lisboa, Portugal; 2 Department Of Psychology, Universitat Rovira i Virgili, Tarragona, Spain; 3 Gift Institute Of Integrative Medicine, Psychosomatic Center, Pisa, Italy; 4 School Of Psychology And Health Sciences, University of Porto, Porto, Portugal

**Keywords:** adherence, confinement, Risk perception, COVID-19

## Abstract

**Introduction:**

Complete adherence to public health guidelines is essential to reduce the spread of COVID-19. Studies on the factors associated with increased/decreased adherence to these measures have the potential to inform public policies directed at increasing adherence, and thus helping to control the spread of the current pandemic.

**Objectives:**

This study aimed at assessing the demographic and psychosocial predictors of the perceived risk of the COVID-19 and adherence to confinement guidelines during the first mandatory lockdown in Portugal.

**Methods:**

A convenience sample of 430 adults living in Portugal between March 19^th^ and May 2^nd,^ 2020 completed an online survey asking participants about the perceived risk of the COVID-19 and adherence to confinement guidelines. Participants also completed a sociodemographic questionnaire and measures of psychological function. Multiple regression analysis was performed.

**Results:**

Teleworking and Risk and COVID-19 controllability were significant predictors of the perceived risk of COVID-19 as measured by the perceived risk of being infected with COVID-19. Teleworking participants and those perceiving COVID-19 as less controllable reported a higher perceived risk of being infected with COVID-19 than those who were not in telework and perceived COVID-19 as a controllable condition. Adherence to confinement guidelines was predicted by the mental health status and perceived risk of COVID-19. Participants who reported worse mental health status, who perceived COVID-19 as a dangerous condition, and who trusted the public health system reported greater adherence to confinement guidelines.

**Conclusions:**

The results of this study will be discussed considering their implications to public health policymaking to promote adherence to public health policies.

**Disclosure:**

No significant relationships.

